# Three-year-olds' theories of mind are symbolic but of low complexity

**DOI:** 10.3389/fpsyg.2014.00682

**Published:** 2014-06-30

**Authors:** Graeme S. Halford, Glenda Andrews

**Affiliations:** ^1^School of Applied Psychology, Griffith UniversityBrisbane, QLD, Australia; ^2^School of Psychology, University of QueenslandBrisbane, QLD, Australia; ^3^School of Applied Psychology, Griffith UniversityGold Coast, QLD, Australia

**Keywords:** theory of mind, symbolic processes, complexity, implicit, explicit knowledge

Rhodes and Brandone (2014) have shown evidence of limited theory of mind in 3-year old children. The study was based on a false belief procedure in which an observer [E1] saw a toy hidden in location 1, then it was moved to location 2. E1 observed the move in the true belief (TB) test but not in the false belief (FB) test. Then the child was assessed for understanding of E1's belief about the toy. In the FB test E1 should falsely believe the toy was still at location 1, whereas in the TB test E1 would know the toy was at location 2. In the action condition the child was asked to open the door where E1 would enter to retrieve the toy, whereas in the verbal condition the child was asked where E1 thought the toy was. Participants performed above chance in both TB and FB tests in the action condition but in the verbal condition they performed above chance in the TB test only. Rhodes and Brandone (2014) interpret correct performance in the action condition as reflecting implicit theory of mind whereas in the verbal condition it is interpreted as explicit.

We accept the empirical findings and agree that the action condition represents a lower level of understanding than the verbal condition. However in their effort to operationalize the implicit—explicit distinction they have also manipulated cognitive complexity. Therefore, we suggest that the study can be interpreted in terms of some deeper theoretical constructs. Specifically, it has recently been proposed that children acquire the beginnings of symbolic processes at approximately 1 year of age (Halford et al., 2013). However, structure is necessary to give symbols meaning, and relations are the essence of structure (Halford et al., 2010). Relational representations are subject to the effect of cognitive complexity, which can be quantified by the number of entities bound in a representation (Halford et al., 2007). Relational representations vary from unary, such as dog (Fido) to binary, such as larger (elephant, mouse), to ternary such as add (3, 2, 5) and quaternary such as proportional (3, 6, 4, 8). Approximate age norms are 1 year, 1½ years, 5 years, and 11 years for unary, binary, ternary and quaternary relations respectively. Theory of mind as assessed in the verbal condition entails ternary relational processes which is one possible reason why success is usually not observed until 5 years, whereas 2-year olds exhibit binary relational theory of mind in the form of connections and transformations tests (Andrews et al., 2003). Our proposal therefore would be that the 3-year olds were employing symbolic processes of low relational complexity.

All participants passed control tests designed to assess their knowledge of: Where the toy was left, where it was after it was moved, and whether E1 had observed the move. These propositions are sufficient for the verbal FB test. Thus if E1 sees the toy at location 1, but does not know it was moved, E1 will (falsely) believe the toy is at location 1. On the other hand if E1 saw the move E1 will know the toy is at location 2. The 3-year olds all answered the three control questions correctly (albeit only on the second test in some cases). So the question is why, given that they had the relevant knowledge, did the children not demonstrate theory of mind in the verbal condition? We suggest that the reason is the cognitive complexity of the verbal FB task, as demonstrated by Andrews et al. (2003). The false belief test entails relating three variables; where the toy was hidden first, the nature of the transformation, and the observer's representation of the toy's location. This is ternary relational and successful performance was associated with other ternary relational tasks including transitive inference and class inclusion. Therefore, it would be expected that 3-year olds, even though they correctly answered the three control questions, would not succeed in the verbal false belief test because they could not construct a cognitive representation that integrated the three relevant variables.

Why then could they pass the action false belief test? We propose that the action test could be passed by the simpler process of representing where E1 last saw the toy. In the FB test E1 last saw the toy at location 1, and therefore will go there to retrieve it. However, in the TB test E1 last saw the toy at location 2, and therefore will attempt to retrieve it there. Both these responses would yield correct scores, and the proportions correct indicate children recognized a link between E1's movement (which door E1 will enter by) and where E1 last saw the toy. Notice however that it does not unequivocally demonstrate that children recognize E1's awareness of the toy's location. Either way, it can be performed by the child's representing a relation between the location where E1 last saw the toy and either E1's retrieval action, or E1's representation of the location:

Child's representation (E1 last-seen location; E1 retrieval-action)

Or

Child's representation (E1 last-seen location; E1 representation-of-location)

In either form this is a binary relation which is easily within the processing capacity of typical 3-year olds (Andrews et al., 2003).

An explanation based on the verbal nature of the explicit false belief test is implausible because the children passed the control questions which were also verbal. It also begs the question why verbal processes are harder. Our suggestion is that relational complexity affects both reasoning and language (Andrews et al., 2006) so both verbal and action performances will be subject to complexity effects. The question: “Where does E1 think the toy is?” would elicit a more complete representation that more closely resembles the representation elicited by traditional false belief tasks in which three variables are related:

Child's representation (where E1 saw toy hidden; nature of transformation; E1's representation of location).

We agree that the action performance is consistent with implicit cognition, but we also suggest it is symbolic, and it is performed correctly because it is structurally simple. Relational complexity has been found to be a powerful explanatory concept in infant, child, adult and animal cognition (Halford et al., 2007, 2014). In the context of theory of mind it implies that implicit (action) theory of mind should be related to many other tasks of similar complexity, including in other domains such as the balance scale and categorical syllogisms. Explicit theory of mind should be related to other ternary relational tasks such as transitive inference and class inclusion.

## Conflict of interest statement

The authors declare that the research was conducted in the absence of any commercial or financial relationships that could be construed as a potential conflict of interest.
